# Photonic reservoir computing for nonlinear equalization of 64-QAM signals with a Kramers–Kronig receiver

**DOI:** 10.1515/nanoph-2022-0426

**Published:** 2022-10-19

**Authors:** Sarah Masaad, Emmanuel Gooskens, Stijn Sackesyn, Joni Dambre, Peter Bienstman

**Affiliations:** Photo-nics Research Group, INTEC Department, Ghent University – imec, Ghent 9052, Belgium; IDLab, INTEC Department, Ghent University – imec, Ghent 9052, Belgium

**Keywords:** Kramers–Kronig receiver, nonlinearity mitigation, photonic reservoir computing

## Abstract

Photonic reservoirs are machine learning based systems that boast energy efficiency and speediness. Thus they can be deployed as optical processors in fiber communication systems to aid or replace digital signal equalization. In this paper, we simulate the use of a passive photonic reservoir to target nonlinearity-induced errors originating from self-phase modulation in the fiber and from the nonlinear response of the modulator. A 64-level quadrature-amplitude modulated signal is directly detected using the recently proposed Kramers–Kronig (KK) receiver. We train the readout weights by backpropagating through the receiver pipeline, thereby providing extra nonlinearity. Statistically computed bit error rates for fiber lengths of up to 100 km fall below 1 × 10^−3^ bit error rate, outperforming an optical feed-forward equalizer as a linear benchmark. This can find applications in inter-datacenter communications that benefit from the hardware simplicity of a KK receiver and the low power and low latency processing of a photonic reservoir.

## 1 Introduction

**Figure 1:**
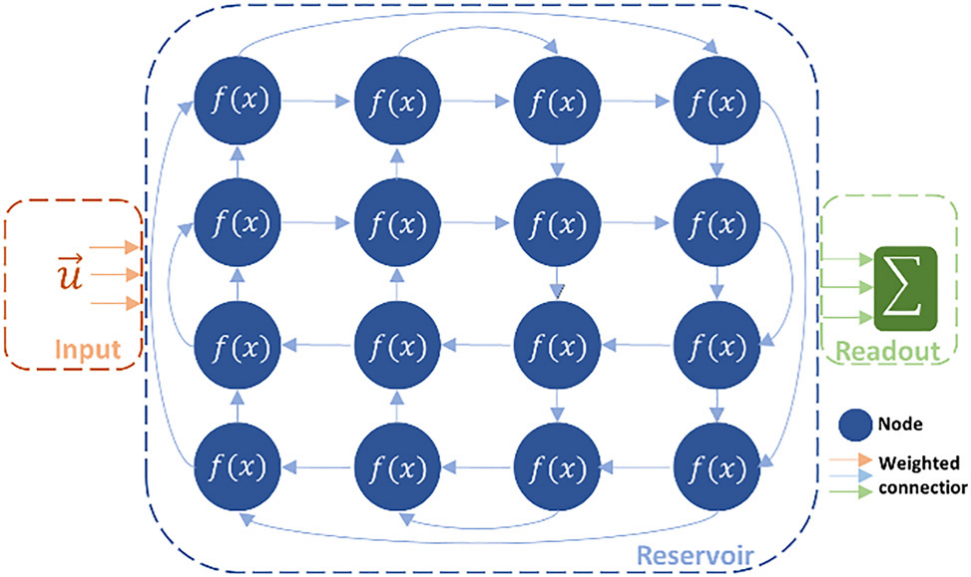
RNNs are networks with feedback connections where all connections are trained. For RC, the network is split into a reservoir layer where connections are untrained, and a readout layer with trainable connections. The readout signals, which can be taken from any and all the reservoir nodes, are summed to generate the output.

Machine Learning (ML) models are ubiquitously used for solving problems across research areas. Often however, powerful models are limited due to their difficult training and high power consumption. Recurrent Neural Networks (RNNs), for example, feature feedback connections that make them well-suited for temporal problems but complicate their training. To this end, Reservoir Computing (RC) has emerged as an alternative for training RNNs [1]. Rather than considering a fully trainable RNN, as seen in Figure 1, the reservoir consists of randomly weighted connections that are left unchanged during training. Only the outer layer weights, collectively known as the readout, are optimized. While RC has fewer free parameters during training, it remains strongly capable of solving machine learning tasks. Moreover, these weights can be found by solving a convex linear function, circumventing the need for iterative methods.

Reservoirs are also advantageous for non-digital computing [2] where physical systems are used as computing substrates. This form of alternative computing is gaining considerable momentum, driven partly by the faltering performance of the conventional Von Neuman structures when training neural networks [3]. A reservoir can be created from a dynamic system by defining function-performing *nodes* that leverage the system’s natural nonlinearities. The nodes are connected through weighted *interconnections* that are tolerant to the inherent manufacturing variations or natural uncertainty present in hardware. These physical reservoirs are leveraged for performing machine learning tasks and are implementable in different substrates, anywhere from mechanical to biological systems [4]. Of significant importance are photonic-based reservoirs, which utilize optical modules to manipulate light [5]. Indeed, the maturing discipline of photonics boasts high bandwidths, allows parallelism through wavelength multiplexing, can be deployed as an integrated circuit, and enjoys optoelectronic interfacing technologies [6]. Moreover, both linear and nonlinear operations can be performed, the former at no additional power costs and the latter occurring inherently in many optical components.

An important propeller for the advancement of photonic reservoirs is their easy integration in fiber optic communication systems to perform optical signal processing. Traditional Digital Signal Processing (DSP) methods suffer from high power consumption and unimpressive performance for some of the challenging problems, including nonlinearity equalization. Although digital-based ML solutions, including neural networks, are also investigated for improved performance [7–10] they do not address the power, memory, and latency issues of electronics. Thus, a photonic reservoir can be added to the processing pipeline to target some of the fiber impairments, offloading parts of the electronic DSP into the optical domain with the aim of eventually migrating to fully optical-based signal equalizers. Initial successes were reported on the use of passive photonic reservoirs on an integrated chip for dispersion compensation and nonlinear equalization of intensity modulated signals in simulations [11] and in experiments [12]. Another integrated reservoir architecture comprising ring resonators showed successful equalization numerically [13]. Time-delay photonic reservoirs [5] have also shown good transmission equalization in simulations [14, 15] and experiments [16]. In [17], a photonic neural network, rather than a reservoir, was experimentally shown to mitigate transmission nonlinearities.

In this work, we target nonlinearities in coherent optical communication systems deploying quadrature-amplitude modulation (QAM). Through modulation of both the amplitude and phase of light, higher data rates are achieved at modest electronic bandwidth requirements. To simplify the detection process, which is otherwise hardware-demanding, the recently proposed Kramers–Kronig (KK) receiver [18] is used. The KK receiver boasts a simple hardware implementation consisting of a single photodiode, and performs the phase reconstruction of the complex signal by leveraging the well-known KK relations. Its hardware simplicity addresses cost concerns associated with coherent detection in short to medium-length links. While there are concerns on the electronic bandwidth requirements to perform the processing required post-detection, this receiver has been gaining popularity due to its hardware simplicity and accurate signal reconstruction compared to other schemes [19]. Several experimental setups were recently demonstrated in optical high-capacity transmissions [20–22] and even in wireless THz communications [23]. An important system consideration is that a high-power subcarrier must be added to the signal at the receiver or the transmitter. For complexity and cost constraints, the latter is preferred but may give rise to high nonlinear effects in the transmission fiber.

To this end, we numerically simulate the use of an on-chip photonic reservoir for the nonlinear equalization of a high intensity 64-QAM signal propagating in a standard single mode fiber (SSMF) spanning lengths of up to 100 km and received by a KK receiver. Nonlinearities originating from the fiber Kerr effects and the transmitter’s nonlinear behavior are considered. In contrast to classical RC which computes the readout weights using ridge regression, our equalizer uses backpropagation through the receiver pipeline to train the optical readout weights. This readout optimization is done to leverage the receiver’s nonlinearity to our advantage which, as will be shown, provides better performance. Furthermore, the reservoir uses 16 readout weights which are trained as single units, rather than separating them into real and imaginary weights, using a complex-input real-output loss function. The test results per data point are found for over half a million bits generated using a Winchman–Hill random generator, and testing errors reported fall well below the 1e-3 pre- forward error correction (FEC) bit error rate (BER) [24]. This implementation can easily cope with higher data rates, unlike electronic solutions which would require higher sampling operation rates. Indeed, as will be shown later, the main adjustment required for higher data rates would be using shorter waveguides, which is both more power efficient and has a smaller chip footprint. As such, it is a powerful solution for mitigating communication system errors, which along with the KK receiver can be viable solutions for the deployment of high data rate and high modulation format systems for inter- and intra-data center applications.

The remainder of this paper is organized as follows: in Section 2 the photonic reservoir architecture used in these simulations is presented along with some background on reservoir computing. In Section 3, the working principle of the KK receiver is outlined. In Section 4, the system design and simulation results are discussed. Finally, the paper is concluded in Section 5.

## 2 Photonic reservoir computing

Figure 1 shows both an RNN and an RC system, as they are both recurrent structures that differ mainly in their training degrees of freedom. Assuming discrete time signals, and using vector notations to denote several such signals, an input $\vec{u}\left[n\right]$ is injected to the network at a subset of the nodes. Every node’s output is given by the node’s transformation function on its inputs. These inputs can either come from within the reservoir or from the input sources. As such, the reservoir’s internal states evolve and can be described by [5]

(1) $$\;\vec{x}\left[n+1\right]=f\left(W_{in}\vec{u}\left[n+1\right]+W_{\text{res}}\vec{x}\left[n\right]\right);$$

where *f* is the node’s transformation function, *W*_in_ is a sparse matrix that indicates the weighted connectedness of the input signal to the network, and *W*_res_ is a matrix resembling the weighted reservoir connections. The output of the reservoir, $y_{\text{readout}}\left[n\right]$ , is a weighted sum of ${\vec{x}}_{\text{out}}\left[n\right]$ :

(2) $$y_{\text{readout}}\left[n\right]=W_{\text{readout}}{\vec{x}}_{\text{out}}\left[n\right];$$

where ${\vec{x}}_{\text{out}}\left[n\right]$ refers to the portion of $\vec{x}\left[n\right]$ that is directed to the readout and *W*_readout_ denotes the adjustable readout weights.

**Figure 2:**
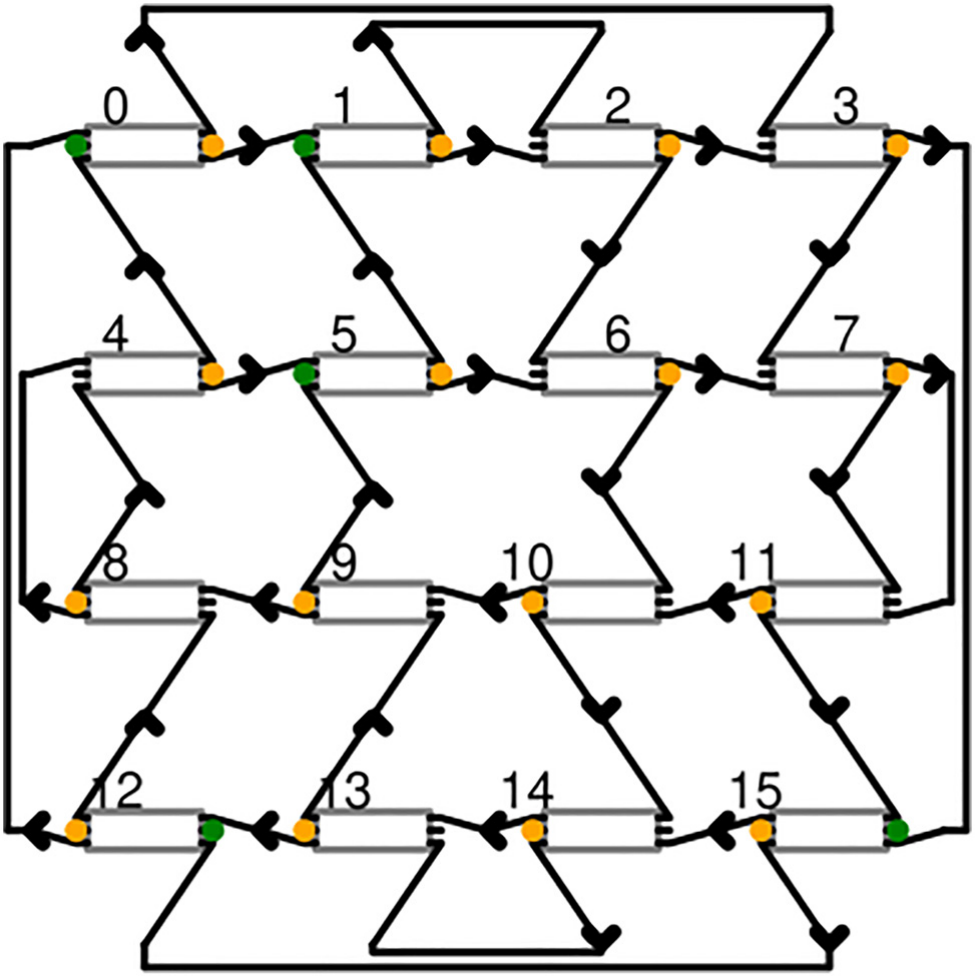
Schematic of the photonic 4-port reservoir. Grey boxes indicate 3 × 3 MMIs, green ports indicate the subset of input injection locations which are to be investigated, and orange dots are where output ports connect to the trainable readout weights. MMIs are connected by waveguides as shown by the black connectors.

Our implementation of the reservoir is a silicon nitride (SiN) based photonic integrated circuit, making it a small-footprint on-chip reservoir. This reservoir, which we term the four-port architecture [25], is made of 3 × 3 multi-mode interferometers (MMI) that serve as nodes and are interconnected by waveguides. A schematic of the reservoir is shown in Figure 2, where the MMIs are indicated by grey rectangular elements and the waveguides are shown as arrowed connectors. Every node has an external input, an external output, two inputs from within the reservoir, and two outputs leading into the reservoir. External inputs are used for injecting signals into the reservoir, where optimal injection locations are investigated in Section 4. The investigated inputs are marked in green in Figure 2. The input weights that impact the signal before its injection to the reservoir manifest in the form of random phase changes on the signal due to waveguide connections from the coupler to the MMI. These are random and unoptimized weights. The MMI outputs are connected to the readout weighting elements where they can be weighted and summed; these are marked in orange in Figure 2. Note that a node may be used to inject a signal, read out a signal, both, or neither. The waveguide delay lines connecting nodes slow down the signal to allow meaningful mixing on the timescales of the input signal. In the silicon nitride platform, the delay lines exhibit typical waveguide losses of 25 dB/m and the MMIs have an insertion loss of 1 dB, both creating the fading memory effect necessary for reservoirs. Without sufficient losses, signals will remain echoing in the reservoir leading to undesired performance.

The waveguide-induced phase and amplitude changes that occur in the reservoir are largely caused by the manufacturing tolerances and imperfections inevitably present in silicon photonics. These act as internal reservoir weights that we do not train nor optimize. We are, however, interested in the length of these lines, as they control the amount of memory in the reservoir and the time differences between two mixing signals. Thus, the delay line length is an optimizable parameter in our setup.

This is a passive reservoir by design; it is driven by the input signal itself and consumes no additional power. It should be noted, however, that the use of the term passive is solely indicative of the need for external power in the reservoir. Currently, due to the lack of more power conservative coupling and photonic components, inevitable losses incurred would require the signal to be amplified. In the SiN chip which this simulation is based on, coupling light into the chip induced around 7 dB loss per coupler. Other losses within the reservoir including insertion losses (1 dB per MMI) and waveguide propagation losses (25 dB/m) are less impactful and are considered necessary for the fading memory of the reservoir. While passive nodes are a power advantage, MMIs perform linear functions that just redistribute portions of the inputs over the outputs, which would not be sufficient to solve nonlinear tasks. The required nonlinear transformation is then added through utilizing innate components in the system where the reservoir is inserted and which may be application specific. In a telecommunication system, a photodetector performing the squaring function is a suitable candidate [26]. Our system is therefore a slight variant of a standard reservoir, where the nonlinearity happens after the readout weights as opposed to within the reservoir. The final output of the reservoir, $y_{\text{final}}\left[n\right]$ , is then the nonlinear transformation of the readout output

(3) $$y_{\text{final}}\left[n\right]=f\left(y_{\text{readout}}\left[n\right]\right).$$

In the next section, we describe through the introduction of the KK receiver’s algorithm the nature of such transformations.

## 3 Kramers–Kronig receiver

**Figure 3:**
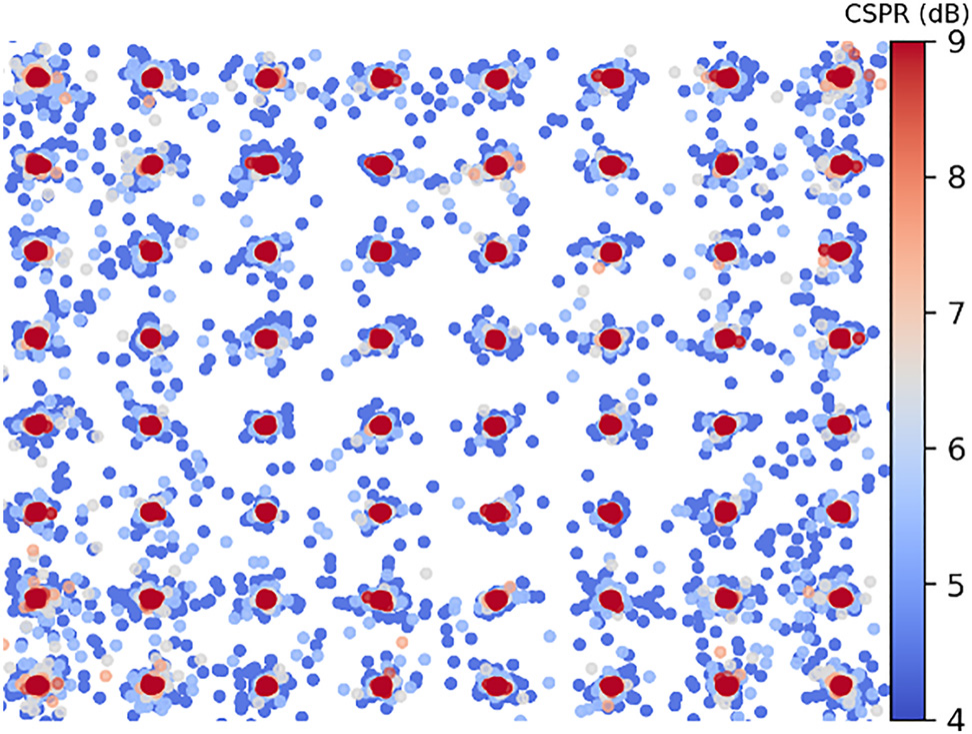
Reconstruction of a 64 QAM signal at different CSPR values. Lower CSPR values (in blue) show large errors, as reconstruction becomes more accurate at higher CSPRs (in red).

The Kramers–Kronig receiver employs a simple photodetector to detect a complex signal’s power and reconstructs its complex nature through a series of processing steps. This is possible when a complex signal’s phase is uniquely related to its amplitude, which can be guaranteed provided that a pair of conditions is respected [27]. First, it must be a single side-band (SSB) signal. Thus, an additional optical subcarrier should be added to either edge of the signal’s spectrum. Second, the subcarrier must have sufficiently higher power with respect to the signal; this is termed the carrier-signal power ratio (CSPR). Figure 3 shows how the reconstruction of a 64-QAM signal is affected by the CSPR in a back-to-back setup. As the CSPR increases, the signal’s reconstruction becomes more accurate (i.e. negligible mean-square error compared to the signal before detection). With compatible signals, the phase information φ(*t*) can be found from the magnitude of the signal $\left|s\left(t\right)\right|$ [18], since

(4) $$\varphi \left(t\right)=\frac{1}{\pi }P\int_{-\infty }^{\infty }\frac{\ln\left(\left|s\left(t\right)\right|\right)}{t-\tau }d\tau$$

where *P* is the Cauchy principal value and ln is the natural logarithm. This equation is based on the well-known KK relations that relate the *real* and *imaginary* part of a signal. Through taking the logarithm of the signal, its *amplitude* and *phase* become separated into the sum of a *real* and *imaginary* part allowing the use of these relations. Indeed, consider the following complex signal,

(5) $$s\left(t\right)=\left|s\left(t\right)\right|e^{j\varphi \left(t\right)}$$

and by taking its natural logarithm becomes

(6) $$\ln\left(s\left(t\right)\right)=\ln\left(\left|s\left(t\right)\right|\right)+j\varphi \left(t\right)$$

**Figure 4:**
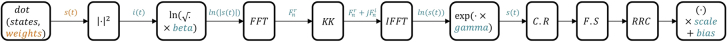
Readout pipeline including receiver blocks, where orange is used for the optical domain and blue for the electronic. Operations – dot, dot product of readout states and optical weights; |·|^2,^ photodetector squaring; ln, natural logarithm; FFT, fast fourier transform; KK, KK relations in frequency domain where frequency coefficients for *f* > 0 are doubled and those for *f* < 0 are zeroed; IFFT, inverse fast fourier transform; exp, exponential; C.R, DC carrier removal; F.S, frequency shifting to the baseband; RRC, root-raised cosine filter; scaling and shifting for constellation alignment signals – $s\left(t\right)$ , complex optical readout signal; *i*(*t*), current; $\ln\left(\left|s\left(t\right)\right|\right)$ , real part of eq. (6); $F_{n}^{r}$ , frequency coefficients of the real signal $\ln\left(\left|s\left(t\right)\right|\right)$ ; $F_{n}^{i}$ , frequency coeffecients of the imaginary signal $j\varphi \left(t\right)$ ; $\ln\left(s\left(t\right)\right)$ , retreived complex signal as in eq . (6); $s\left(t\right)$ , electronic reconstruction of the optical s(*t*). Trainable parameters – weights: complex optical weights; beta, gamma: electronic nonlinearity weights; scale, bias: electronic aligning parameters.

Although this is the basic theory, practical implementations of the KK receiver may circumvent performing time integrals as they can be quite involved. Alternatively, going through the frequency domain and utilizing the Fourier coefficients can be simpler. Some of the detailed implementations are found in [27–29]. Our readout pipeline, including the receiver blocks, is shown in Figure 4. Consider a complex optical signal $s\left(t\right)$ that is impinging on a photodetector. The current from the detector, $i\left(t\right)$ , is the measurable signal obtained and its square root corresponds to $\left|s\left(t\right)\right|$ . The natural logarithm of this quantity is the real part of $\ln\left(s\left(t\right)\right)$ as shown in Eq. (6). A Fourier transform then finds the frequency coefficients of this real-valued signal. From those, the frequency coefficients of the imaginary part, $j\varphi \left(t\right)$ , are computed and the union of both form the Fourier transform of the complex $\ln\left(s\left(t\right)\right)$ [27]. The inverse Fourier transform then generates the time signal which requires exponentiating to retrieve $s\left(t\right)$ .

Figure 4 also shows how the KK receiver is integrated into the readout pipeline so as to serve as the nonlinear transform described by Eq. (3). Although ideally the KK receiver is a linear block [18], it may behave nonlinearly when its minimum phase conditions are violated. This can be seen, for example, in the imperfect reconstruction of the receiver shown in Figure 3 when the CSPR is low. Moreover, while a certain CSPR may be sufficient for accurate signal reconstruction of an undistorted signal, this may not be the case if the signal’s peak-to-average power ratio (PAPR) changes due to dispersion [30]. Other violations including the growth of frequency components in the suppressed sideband will also result in some nonlinear behavior [31]. These violations may become significant when the signal is strongly distorted prior to detection due to dispersion and/or nonlinearities. To further capitalize on the potential nonlinear behavior, electronic weights are added in the receiver pipeline at the natural logarithm and exponent functions, as shown in Figure 4. These are simply trainable electronic weights that are initialized at 1 + 0j values, so as to have no impact in their worst-case performance. The weights are then adjusted through the backpropagation algorithm during the training phase of the readout. Finally, to allow proper alignment with the QAM alphabet, electronic scaling and biasing terms are also included. Together, the optical readout weights, the nonlinearity electronic weights, and the electronic scale and bias form the set of trainable parameters adjusted during the readout training phase. This training is an iterative process, since backpropagation through the receiver blocks is required to train the different parameters.

Following the KK processing, additional steps may be required to obtain the baseband signal. Namely, the additional subcarrier, which will show as a DC component after reconstruction, must be removed. Moreover, the signal should be frequency shifted since it will be offset from its baseband form [29]. Finally, if any matched filtering is used at the transmitter (e.g. a root-raised cosine filter), a similar one should be used at the receiver.

Other practical considerations may be of relevance in the receiver. For example, the logarithmic operation causes spectral broadening and thus would require the input signal to be oversampled. This means that although the Nyquist criterion for the unambiguous signal detection is just 2 samples/symbols, for accurate reconstruction using the KK receiver at least 6 samples/symbols are needed as per the original implementation [18]. Moreover, the issue of the subcarrier and its required power for accurate reconstruction with minimal nonlinearities is a subject of investigation. These and other practical considerations and alternative implementations are discussed in several papers including [20, 29, 32], [33], [34]. In this paper we keep a sufficiently high carrier power and investigate, as will be shown in the following section, the added benefit of the photonic reservoir in mitigating the nonlinearity-induced errors.

## 4 System setup and results

**Figure 5:**
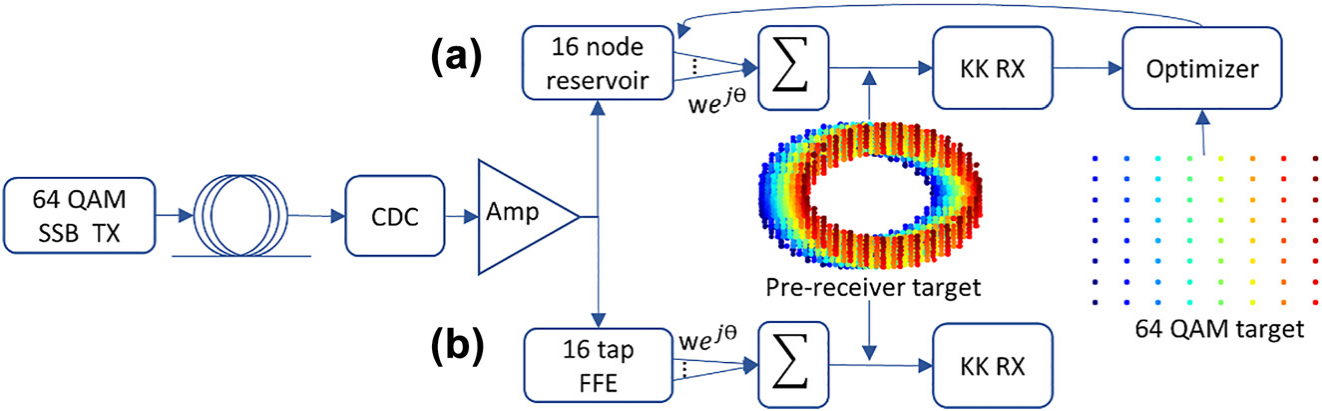
Simulation setup generating high intensity 64-QAM signals passed through fiber links that are dispersion compensated, nonlinearity equalization to take place in: (a) 16 node reservoir with complex readout weights found linearly and through backpropagation; or (b) 16 tap feed forward equalizer with complex weights found linearly. Acronyms–SSB Tx, single-sideband transmitter; CDC, chromatic dispersion compensation; Amp, amplifier; KK RX, Kramers–Kronig receiver; wej^θ^, complex weight with amplitude *w* and phase *θ*.

The transmission system is simulated using VPI Photonics Transmission Maker software [35]. A single polarization transmitter deploying a pair of single-drive Mach–Zehnder modulators (MZMs) is used for 64 QAM transmission. The nonlinear behavior of the modulators is not pre-compensated for. The laser is set to have an average power of 0 dBm before modulation. After modulation, the signal is amplified such that the average power of the signal is 3 dBm. The signal is then noise loaded to 27 dB optical signal-to-noise power ratio (OSNR). A subcarrier is frequency shifted from the main carrier and transmitted with the signal through links between 20 and 100 km of SSMF. The main role of the reservoir is to target nonlinearity-induced errors generated from self-phase modulation due to the Kerr effect and from the nonlinear response of the MZMs. In order to focus on that role, we compensate linear dispersion separately using a dispersion compensation fiber with (negatively) matched dispersion parameters to those of the transmission fiber. The signal is then amplified to correct for the fiber attenuation and adds an additional 15 dB of power. The receiver is the KK receiver pipeline described in the previous section, where the photodetector simulated exhibits shot and thermal noise as described by the parameters in Table 1. No other impairments, including polarization mode dispersion (PMD), laser phase noise and receiver filter imperfections are considered. The system parameters are listed in Table 1 and the setup is shown in Figure 5.

**Table 1:** System parameters.

Transmitter (Tx)	Fiber	Receiver (Rx)
Root-raised cosine filter rolloff = 0.01	Attenuation = 0.02 dB/km	Bandwidth = 70 GHz
Gray-coded 64 QAM	Nonlinear coefficient = 1.31 W^−1^ km^−1^	Samples/symbol = 8
Baud rate = 64 Gbauds	Length = 20–100 km	Responsivity = 0.5 A/W
Signal power (without subcarrier) = 3 dBm	Dispersion parameter = 16 ps/(nm km)	Dark current = 5 nA
Subcarrier power = 14 dBm	PMD coefficient = 0 ps/ $\sqrt{km}$	
Subcarrier frequency offset = 34 GHz		
Laser linewidth = 0 Hz		
OSNR = 27 dB		

A 16-node reservoir, whose internal topography is the same as that shown in Figure 2, was simulated using Photontorch [36]. This is a photonic circuit simulator written in Python that uses adjustable parameters and scatter matrices to describe components. To allow the components parameters to be optimized using iterative methods, Photontorch uses the machine learning framework Pytorch [37].

All the reservoir’s nodes are connected to the readout and the complex-valued optical weights of the readout are trained to approximate the target signal. Initially, the target signal before the KK receiver is used, which is an SSB signal with an additional subcarrier. This results in the rotating target signal shown in Figure 5. The target signal is generated from an ideal transmitter that does not suffer from the nonlinear modulator response and without the noise loading performed for the distorted signal. The same subcarrier generated for the distorted data is used for the target signal, since in both cases no linewidth was considered. It was observed that training to optimize for the center samples of the waveforms yields better performance than training to optimize for the full waveform, and as such is used throughout the training processes. This training is helpful in addressing the out of band noise in the signal and conditions the reservoir to behave as a bandpass filter. Without such filtering, strong violation of the SSB condition is observed due to noise and due to the growth of frequency components from four-wave mixing, which significantly deteriorates the performance of the receiver. Using this signal as a target is beneficial in reducing errors, however suffers from two deficits. Primarily, no nonlinear component was introduced in the pipeline, which limits the nonlinear problem-solving capacity of the reservoir. Additionally, this target is not the final target in the pipeline, and hence optimizing for it would be suboptimal. This method, where the pre-receiver target is used, is referred to as the linear RC (L-RC), in reference to the linear nature of the readout. The final target is only available after the KK DSP and post processing, in which case the undistorted signals are used but without the subcarrier. As seen in Figure 5, these are the standard 64 QAM waveforms. Using this target mandates backpropagating through the receiver blocks to bridge the gap between the readout weights and the final target. This also gives rise to a nonlinear “function” which encompasses the photodiode, the KK algorithm, and the post-processing steps. This is referred to as the non-linear RC (NL-RC) since a nonlinear function is now involved. In spite of more complicated training, through this adjustment a significant error reduction can be obtained as will later be shown in Figure 8.

We will compare our results to a linear baseline, whose pipeline is shown in Figure 5b, utilizing an optical 16-tap feed-forward equalizer (FFE) instead of the reservoir. The FFE passes the signal through a series of 15 cascaded time delays (implemented optically as waveguides). The signals at the output of every delay and a portion of the original signal are weighted with trainable complex-valued weights and then summed. The equalized signal is then sent to the receiver for detection. This is a linear block and would use the target before the receiver for adjusting the weights, similar to the L-RC target. Although the choice of an optical FFE deviates from the standard electric one, we opted for the optical implementation as a closer benchmark to our optical reservoir. Furthermore, if the FFE were implemented digitally, an additional optical bandpass filter prior to detection would still be needed making the direct comparison unfair.

In such data-assisted equalization, relevant system parameters (like the nonlinearity coefficient value) are not needed for adjusting the equalizer. Instead, supervised learning is done to adjust the weights through utilizing a labelled training dataset. Around 50,000 bits are used for training, generated through the random seeding of a Wichmann–Hill number generator, with a cycle length exceeding 2 × 10^13^ [38]. This set is passed through the transmission fiber and, aided with its undistorted version used as a target, the weights are found.

When using a linear readout, as in the L-RC and the FFE, weights can be calculated using a closed-form solution from the input signal matrix *X* and the output target *y*. This is given by

(7) $$W={\left(X^{H}X+\alpha I\right)}^{-1}X^{H}y$$

where *W* is the weight matrix, *α* is a regularization parameter to limit overfitting, *I* is the identity matrix. The superscript *H* refers to the conjugate-inverse of the matrix, which is needed for complex-valued data.

For the NL-RC the weights are trained through backpropagation [39] to minimize a loss function. This training method relies on computing the gradient of the error function with respect to the weights. The gradient calculation begins from the output and propagates in the direction of the input, i.e. moving backwards. The weights are then adjusted in the direction of the decreasing gradient to approach the local minima of the loss function. This is done iteratively over a number of training steps. For training, the machine learning framework Pytorch was used.

The loss function used was the mean-square-error (MSE) loss function with complex valued inputs. This is not inherently supported in Python’s Pytorch, but MSE’s definition can naturally be extended for such data types. The computed loss is then is real-valued and can be supported by Pytorch.

(8) $$loss={\left(\left|y-X\right|\right)}^{2}$$

An issue of the backpropagation algorithm is that it may get trapped in high-energy local minima. To avoid this, we initialize the weights with well-chosen initial values prior to backpropagation, as opposed to starting from a random initialization. For these initial weights we use those of the closed form solution, i.e. the L-RC. The backpropagation then starts from this point and then iteratively adjusts the weights to approach a lower loss value.

**Figure 6:**
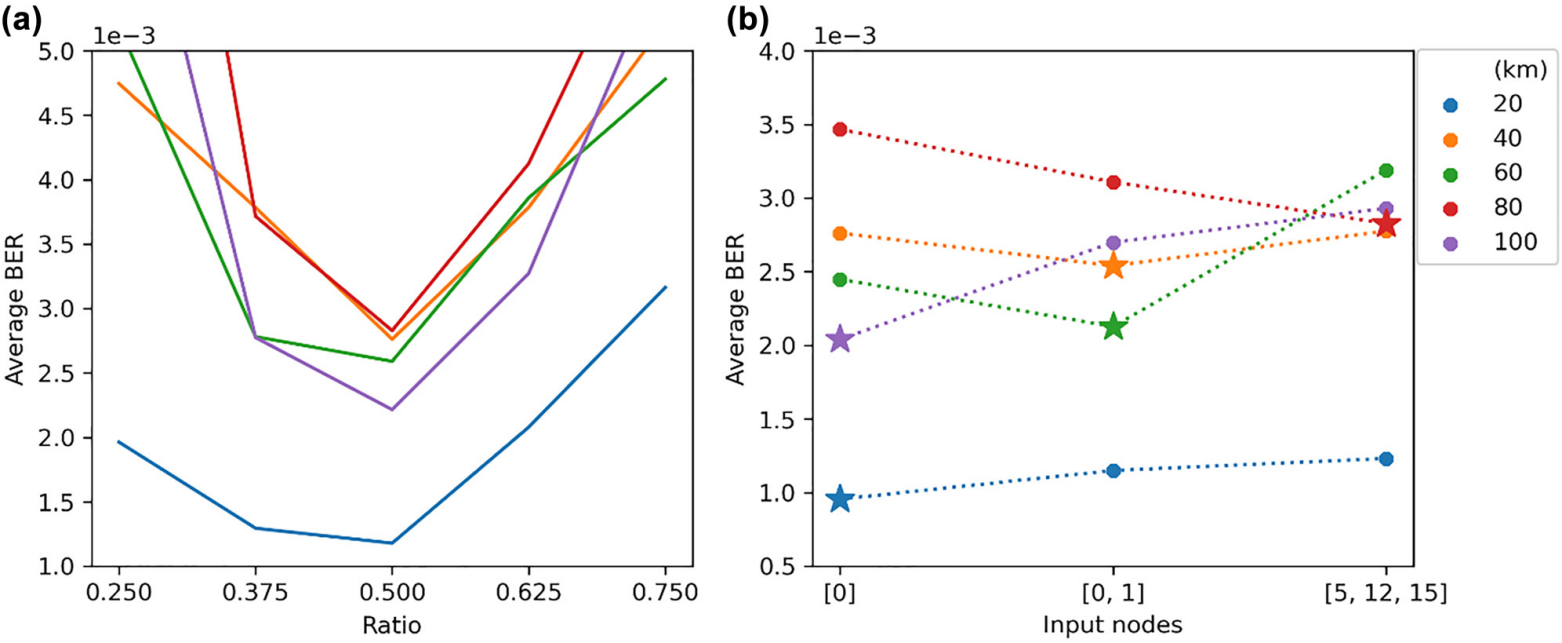
Optimization of reservoir parameters using BER metric for all link lengths individually, two parameters are studied: (a) (Left) the interconnection delay expressed as a ratio of the delay induced in seconds to the symbol rate of the signal. (b) (Right) the nodes used to inject the signal into the reservoir.

The performances of both the reservoir and the FFE are reliant on their configurable parameters. For the reservoir, this includes the length of the delay lines connecting the nodes, as well as the choice of nodes that the input is injected at. For the FFE, only the delay line length is relevant. Thus, the performance of different configurations is compared by sweeping through the optimizable parameters, finding the weights, and then using them to calculate the achievable BER. Since this is part of the optimization process, the training dataset is used to guide the process (later on we will use a separate testing set for the final performance evaluation). Furthermore, the optimization of the reservoir parameters is done based on the performance of the L-RC weights since backpropagation is a time-costly process. The underlying rationale is that the best performing configuration for the L-RC is very likely to be the best one for the NL-RC. The results of the architecture optimizations are shown in Figure 6.

Initially, the interconnection length is investigated (Figure 6a), where this refers to a single value used to specify the length of all the reservoir’s node to node connections as shown in Figure 2. To choose the best length, we sweep over values expressed as a ratio of the induced delay (in time) to the baud rate of the signal. Since the reservoir interconnections contain inherent phase uncertainties because of the waveguide’s sidewall roughness, it is important to ensure that the performance is consistent over multiple random reservoirs. The waveguide variations are modelled in simulations as a phase shift of 2*πr* *,* where *r* is a random number sampled from a uniform distribution between 0 and 1. Every waveguide in the reservoir is assigned a different random phase shift and these vary from one reservoir initialization to another. Furthermore, the input signals to the reservoir are assigned random phase shifts governed by the same distribution as above. Thus, every datapoint is the averaged performance of several different reservoirs each with a separate random (but static) set of interconnection phases. Moreover, since every fiber length constitutes a different problem, its optimization is done independently. However, optimizations for all fiber lengths show that the best performance is achieved around a ratio of 0.5. The dependence of this ratio on the data rate of the signal indicates that higher data rates would require shorter wave guides to maintain the same ratio. Thus, such a reservoir would scale well with higher baud rates since the required shorter waveguides will have lower losses and a smaller footprint.

Next, we look into the effect of the choice of input nodes on the performance. Referring to Figure 2, nodes are assigned numbers from 0 to 15 from left to right (the numbering continues from the left at every new row). All nodes, and any subset of them, can be used to inject the signal into the reservoir which affects how they mix. To ensure fair comparison, the signal power is divided equally between the input nodes, i.e. the total power injected in the system is kept constant, regardless of the number of inputs. Three input configurations are contrasted, which are indicated on the *x*-axis of Figure 6b. The reported BER of the training set is again averaged over multiple random initializations of the reservoir. The best performing input configuration for every link length is indicated by a star whereas all the other suboptimal configurations are indicated by a dot.

**Figure 7:**
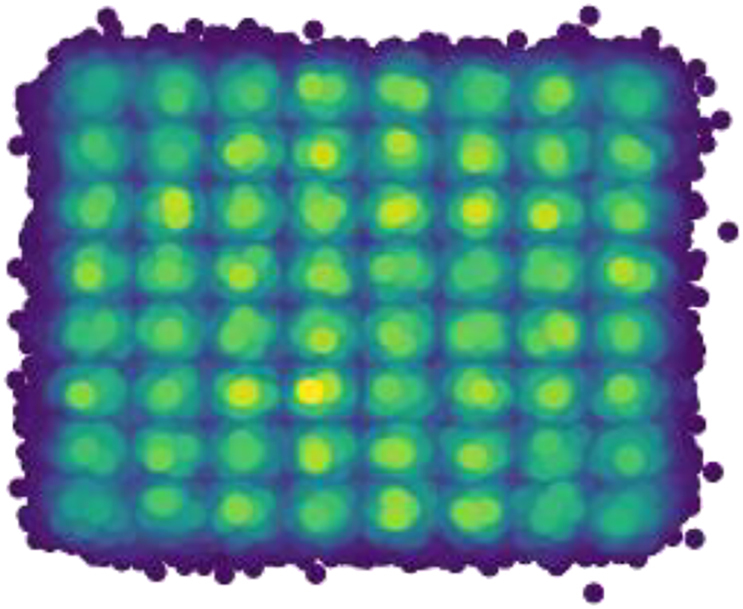
Density distribution of a linearly and nonlinearly distorted 64-QAM showing the highly distorted signal.

Based on the results shown in Figure 6, the optimum architecture parameters for every fiber link are chosen. This is also done for the FFE where its delay line length is optimized for every fiber link length. The length of the delay lines governs the delay of signals with respect to one another and as such it also governs the points at which the signals are summed. Then, for each of these optimized architectures, the training dataset is used to find the trainable weights. Finally, a testing dataset is generated to investigate the performance of the equalizers. The unseen testing set is generated through the same Wichmann–Hill generator but using different seeds to that of the training set. Over 130,000 symbols are tested and the statistical BER is found using a gaussian approximation. To illustrate the difficulty of the problem, Figure 7 shows an example of the detection of a distorted signal (after chromatic dispersion compensation). The high-power subcarrier induces nonlinearities to which the high-level modulation format becomes very susceptible. Additionally, due to the growth of unwanted components in the suppressed sideband of the subcarrier, the receiver conditions are violated contributing to the high errors post-detection. As a result, the BER is on the order of 1e-2.

**Figure 8:**
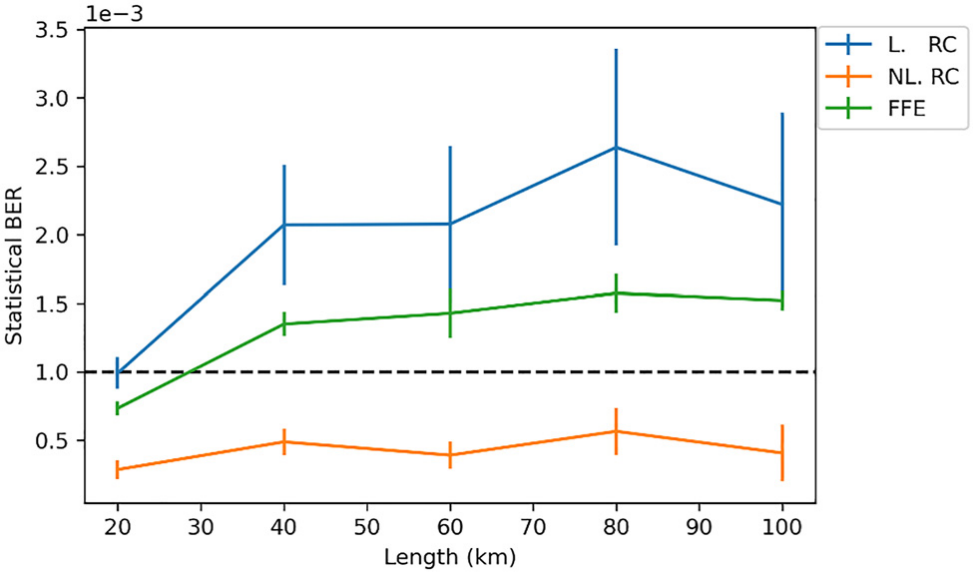
Testing BER versus link length for linear reservoir (blue) where no external nonlinearity is present, nonlinear reservoir (orange) where the KK receiver is leveraged as a nonlinear block, and an optical feed forward equalizer (green) for benchmarking.

Figure 8 shows the averaged statistical BER on the test set for the different fiber lengths. The statistical errors are computed using VPI’s error estimators which assume that the probability of receiving a data point belonging to a QAM symbol follows a gaussian distribution. The probability that the symbol will be in error is computed statistically using the standard deviation and mean of the points. This provides the symbol error rate while the BER is found by dividing by 6 (i.e. the number of bits per symbol). The statistical errors are very close to the errors found by error counting since the test set is rather large. Linear solutions, i.e. FFE and L-RC, improve the BER by means of filtering and by utilizing information from neighboring symbols since they behave as optical linear filters. While filtering improves the performance of the receiver reconstruction approximating that of an ideal coherent receiver, the linear mixing also contributes to reducing errors by utilizing any linear information that is relevant from neighboring symbols. However, the excessive mixing in the reservoir does not directly have a positive impact as compared to that of the FFE. It is only when the nonlinear readout is involved that the BER improves and is on average one third that of the FFE. The performance of the NL-RC for all the fiber lengths reduced the BER well below 1e-3 and maintained this performance with different reservoir simulations and datasets, as indicated by the error bars. The error bars are derived as the standard deviation of 10 results obtained by evaluating the performance of a random reservoir (generated as described for the random initializations of Figure 6), trained and tested on different and randomly generated data sets.

## 5 Conclusions

The transmission of a 64 QAM signal and its detection using the KK receiver was shown to benefit from the use of a photonic reservoir to mitigate the effects of the fiber nonlinearities and the transmitter imperfections. We used a novel training scheme, which included the entire KK processing pipeline to increase the nonlinear computational capacity of the setup. This optical solution is passive, process signals in real time, and future-proofed to operate at high baud rates which makes it superior to other electronic solutions. Simulation results showed that the reservoir outperformed a linear feed-forward equalizer implemented optically, thus displaying beneficial nonlinear functionality. Statistical bit error rates were obtained on testing sets of over 130,000 randomly generated symbols for link lengths of up to 100 km and were well below the 1e-3 threshold. Future work will target the integration of this solution with the well-established digital signal processing to evaluate its performance with additional system impairments like phase noise.
